# Modeling habituation of auditory evoked fields using neural mass models

**DOI:** 10.1186/1471-2202-12-S1-P368

**Published:** 2011-07-18

**Authors:** Peng Wang, Thomas Knösche

**Affiliations:** 1 Max Planck Institute for Human Cognitive and Brain Sciences, Leipzig, Germany

## 

**Figure 1:**
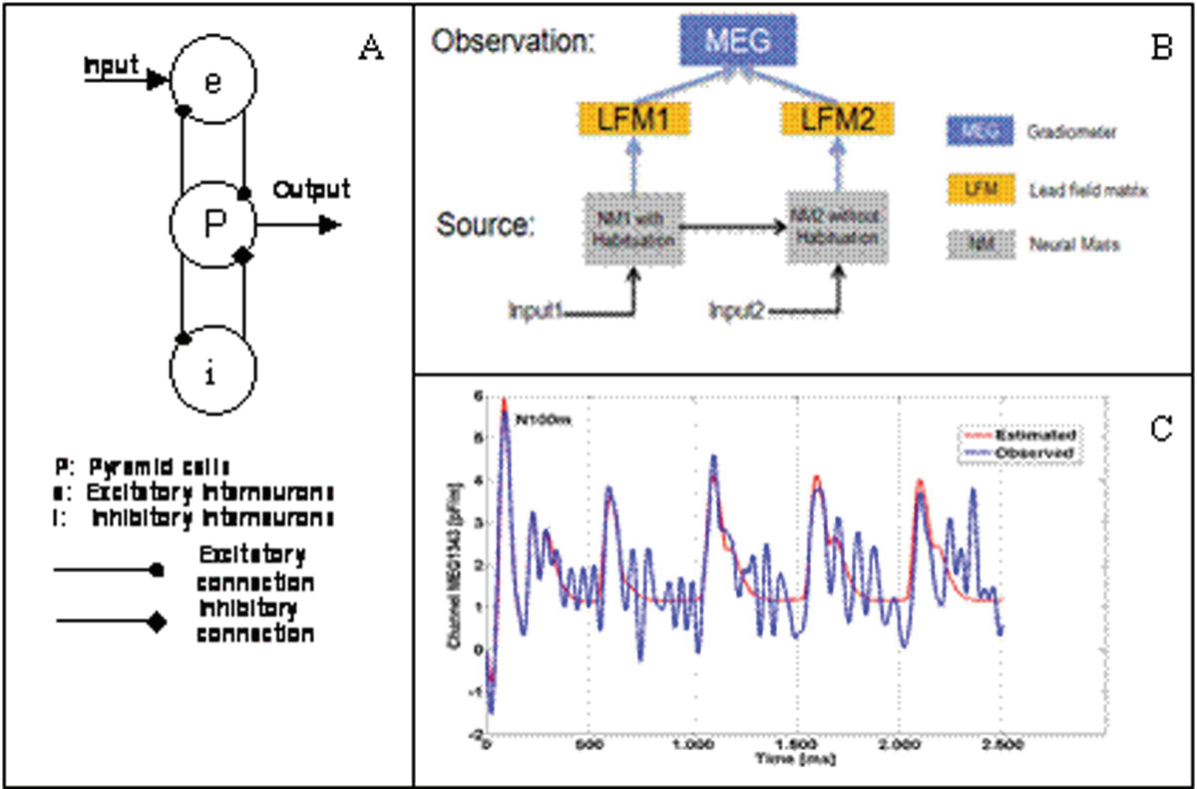
(A) Architecture of a single neural mass model. The output is the average depolarization of pyramidal cells. (B) Architecture of the observation model. The measured signal is assumed to be equal to the source output multiplied by the lead field matrix. (C) Observed and estimated signal.

In the auditory modality, repetitions of stimuli with short time interval usually lead to a decrease of the auditory evoked potentials/fields, in particularly its N100 component [1]. One generally accepted explanation of such short-term habituation is the decrease of the synaptic efficiency [2]. Based on this, we present a mathematic model for this phenomenon: (1) we used two hierarchically coupled neural mass models (NMMs) (Fig. 1A) [3] to account for the basic shape of the auditory cortical response; (2) we implemented a learning rule to modify the excitatory intrinsic connections between the neuronal populations to phenomenologically explain the decrease of the auditory response as a function of the stimuli repetition: *w* = –*n*_1_(*q*/*q*_max_)*w* + *n*_2_(1 – *w*), where *w* (0<*w*<1) is the synaptic connection efficiency, *q* is the incoming presynaptic firing rate, *q*_max_ denotes the maximally possible firing rate, and *n*_1_ and *n*_2_ govern the habituation and recovery rates. The habituation process is assumed to take place at the primary auditory cortex (first NMM). Spontaneous recovery works both in presence and absence of input. The model parameters were estimated from empirical MEG data using Bayesian inference [4]. In order to link the model to MEG, a lead field matrix (LFM) [5] is necessary (Fig. 1B). For each NMM an equivalent dipole was used to locate the anatomical source position for the LFM calculation. Dipole fitting and LFM calculation were performed with the MNE tool box v2.7.2. In our pilot study this model yielded a reasonable fit of the data (N100 peak) (Fig. 1C).

With our habituation model we were able to fit the auditory N100 component and its decreasing amplitude during receptive stimulation. With this simple model, we were not able to account for the later components. The noise had a strong effect on the later components and in order to keep the balance between the model complexity and the fitting we chose only two columns to model the whole ERP. However, our model can be easily expanded to explain details such as P200, P300 etc., by using more NMMs, if more or better quality data are available.
